# Mechanism of microRNA regulating the progress of atherosclerosis in apoE-deficient mice

**DOI:** 10.1080/21655979.2021.2004979

**Published:** 2021-12-02

**Authors:** Xiaoqian Lou, Dawei Wang, Zehui Gu, Tengteng Li, Liqun Ren

**Affiliations:** a Department of Experimental Pharmacology and Toxicology, School of Pharmacy, Jilin University, Changchun, Jilin, China; b Department of Endocrinology, The First Hospital of Jilin University, Changchun, Jilin, People's Republic of China; c Department of Emergency, The First Hospital of Jilin University, Changchun, Jilin, People's Republic of China; d Department of Pathology, The Third Affiliated Hospital of Jinzhou Medical University, Jinzhou, People's Republic of China

**Keywords:** MicroRNA, Atherosclerosis, Apoe-deficient mouse, Gene regulation, Bioinformatic analysis

## Abstract

MicroRNAs play important roles in atherosclerogenesis and are important novel pharmaceutic targets in atherosclerosis management. The whole spectrum of miRNAs dysregulation is still under intense investigation. This study intends to identify more novel dysregulated microRNAs in atherosclerotic mice. Half of eight-week-old male ApoE-/- mice were fed with high-fat-diet for 12 weeks as a model mice, and the remaining half of ApoE-/- mice were fed with a normal-diet as a control. A serum lipid profile was performed with ELISA kits, and atherosclerotic lesions were assessed. Aortic tissues were dissected for gene expression profiling using a Multispecies miRNA 4.0 Array, and significant differentially expressed miRNAs were identified with fold change ≥ 2 and p < 0.05. Real-time quantitative PCR was used to validate microarray gene expression data on selected genes. Predicted target genes were extracted and subjected to bioinformatic analysis for molecular function and pathway enrichment analysis. Model mice showed a 15.32% atherosclerotic lesion compared to 1.52% in the control group. A total of 25 significant differentially expressed microRNAs were identified, with most of them (24/25) downregulated. Real-time quantitative PCR confirmed the GeneChip data. Bioinformatic analysis of predicted target genes identified high involvement of the PI3K/Akt/mTOR signaling pathway. Microarray profiling of miRNAs in high-fat-fed Model mice identified 25 differentially expressed miRNAs, including some novel miRNAs, and the PI3K/Akt/mTOR signaling pathway is highly enriched in the predicted target genes. The novel identified dysregulated miRNAs suggest a broader spectrum of miRNA dysregulation in the progression of atherosclerosis and provide more research and therapeutic targets for atherosclerosis.

## Introduction

Atherosclerosis is well known as the leading cause of human mortality [1,2]. The development of atherosclerosis is a chronic inflammatory process involving pathological changes in vascular endothelial cells [3,4], monocytes/macrophages [5,6], and vascular smooth muscle cells [7,8]. Beyond those identified risk factors, including hypertension, hyperlipidemia, diabetes, inflammation, and oxidative stress [9–13], the fundamental mechanisms are still under extensive investigation.

MicroRNAs (miRs) are short non-coding RNAs (~ 22nt in length), which regulate gene expression by degrading target mRNAs and/or inhibiting their translation and have important roles in atherosclerogenesis [14–18]. Wide ranges of dysregulated miRNAs identified from different sources, like serum, atherosclerotic tissue, endothelium cells, vascular smooth muscle cells, and subsets of macrophages, exert broad pathological effects on atherosclerogenesis through effects on endothelial cells [19–22], inflammatory cells/macrophages [23–26], vascular smooth muscle cells [27–31], and lipid metabolism [32–34]. MicroRNAs are new biomarkers and potential therapeutic targets in atherosclerosis. ApoE knockout (ApoE-/-) mice have been widely used as an atherosclerotic animal model to identify and test dysregulated miRNAs during atherosclerogenesis. Different sets of dysregulated miRNAs were identified by researchers using ApoE-/- mice fed with a Western-type diet (21% fat). Like Han [35] identified 75 differentially expressed miRNAs from pooled atherosclerotic aortic tissues of 8-month-old ApoE-/- mice, and Zhen [36] identified 9 (at 8 weeks) and 19 (at 20 weeks) differentially expressed miRNAs at different stages from atherosclerotic aortic tissues. ApoE-/- mice fed with a high-fat diet (40% fat) show early and extensive atherosclerosis lesions, and various dysregulated miRNAs are reported, such as miRNA-155 [37], miRNA-26a-5p [38], miR-6931-5p, mmu-miR-3547-5p, mmu-miR-5107-5p, mmu-miR-6368, and mmu-miR-7118-5p in aorta atherosclerotic lesions [39]. The continuous identification of novel dysregulated miRNAs suggests dynamic regulation of miRNAs in the progress of atherosclerogenesis, but the whole spectrum of miRNA regulation during the process of atherosclerosis is still unclear. This study intends to identify more novel dysregulated microRNAs in atherosclerotic ApoE-/- mice. The aim is to provide more research and therapeutic targets for atherosclerosis. We report here additional novel dysregulated miRNAs in ApoE-/- mice fed with a high-fat diet for 12 weeks and bioinformatic analysis on predicted target genes.

## Materials and methods

### Animal model establishment

Eight-week-old male ApoE homozygous deficient (ApoE^-^/^-^) mice were obtained from HuaFuKang Bioscience (Beijing, China). Half of the ApoE-/- mice were used as a control and fed with a regular chow diet, and the remaining half of ApoE^-^/^-^ mice were fed with a high-fat diet containing 40% kcal fat (cocoa butter and soybean oil) and 1.25% kcal cholesterol (HFD, D12108C, Research Diets Inc.) for 12 weeks. This study was approved by Jilin University Laboratory Animal Ethics Committee (Changchun, China), and all animal-related procedures were performed in accordance with Chinese regulations on the use of laboratory animals.

### Serum lipid profile

Blood samples were collected from the eyeballs after administering anesthesia, and the serum was isolated by centrifugation at 2500 rpm for 15 minutes. Total cholesterol (TC), triglyceride (TG), high density lipoprotein cholesterol (HDL-C), low density lipoprotein cholesterol (LDL-C), and oxidized low density lipoprotein (ox-LDL) levels were determined using commercial ELISA kits acquired from Nanjing Jiancheng Bioengineering Institute (Nanjing, China).

### Atherosclerotic lesion assessment

The entire aorta (from the aortic root to the iliac artery) was dissected after initial perfusion (cold 1× phosphate buffer saline) through the left ventricle. For atherosclerotic lesion assessment, the section between the aortic arch and the common iliac artery was fixed in 4% paraformaldehyde for 24 hours and subjected to Oil Red O-staining as described [40]. After staining, the aorta was longitudinally opened to visualize the atherosclerotic lesions stained in red. Digital photos were taken using a digital camera (Canon EOS80D, Tokyo, Japan) and subjected to image analysis using Image-pro Plus 6.0 software (NIH Image, USA). The area of the atherosclerotic lesions was calculated as a percentage of the Oil Red O positive area. Aorta specimens were also fixed with buffered formalin solution and subjected to H&E staining.

### MicroRNA microarray analysis

The whole aorta tissues (3 in each groups) dissected free of surrounding tissues were washed with diethyl pyrocarbonate-treated phosphate buffer saline and snapped frozen in liquid nitrogen. The total RNA was isolated with TRIzol reagent (Invitrogen, Carlsbad, Canada) combined with RNeasy Mini Kit (Qiagen, Hilden, Germany). MicroRNA profiling was performed using the Multispecies miRNA 4.0 Array (Affymetrix GeneChip®, USA) at the service of CNKINGBIO (Beijing, China). Briefly, 130 ng of total RNA was used for cRNA synthesis, and the cRNA was hybridized for 16 hours at 45°C following fragmentation. GeneChips were washed and stained using Affymetrix Fluidics Station 450 and then scanned using GeneChip® Scanner 3000 7 G.

### Differentially expressed microRNAs

The microarray data were first analyzed using microarray analyze software at the service of CNKINGBIO (Beijing, China) based on a median summarization normalization method and the random variance model t-test. The differentially expressed miRNAs were extracted with p-values lower than 0.05 and a fold change of at least 2. Differentially expressed miRNAs were subjected to further analysis using Cluster 3.0 and Treeview v1.60.

### Real-Time Quantitative Polymerase Chain Reaction (RT-qPCR)

RT-qPCRs were performed on eight differentially expressed microRNAs to verify the GeneChip analysis data. Briefly, first-strand cDNA Synthesis was done using M-MLV Reverse Transcriptase (Promega, WI, USA), and RT-qPCRs were performed using SYBR Green kit (Takara, Dalian, China) on ABI 7500 real-time PCR system (Applied Biosystems, Foster City, CA, USA). The normalized relative gene expression level was calculated based on the 2^-ΔΔCt^ method [41] using 18S RNA as an endogenous control. The primers used for RT-qPCR are listed in Table 1.

**Table 1.** Primer sequences used for qRT-PCR

Gene	primer (5’→3’)
mmu-miR-322-5p	RT Primer: GTCGTATCCAGTGCAGGGTCCGAGGTATTCGCACTGGATACGACTCCAAA Forward: GCGCAGCAGCAATTCATGT
mmu-miR-674-3p	RT Primer: GTCGTATCCAGTGCAGGGTCCGAGGTATTCGCACTGGATACGACTTGTTC Forward: GCGCACAGCTCCCATCTCA
mmu-miR-30 c-2-3p	RT Primer: GTCGTATCCAGTGCAGGGTCCGAGGTATTCGCACTGGATACGACAGAGTA Forward: CGCTGGGAGAAGGCTGTT
mmu-miR-30e-3p	RT Primer: GTCGTATCCAGTGCAGGGTCCGAGGTATTCGCACTGGATACGACGCTGTA Forward: GCGCTTTCAGTCGGATGTT
mmu-miR-208a-5p	RT Primer: GTCGTATCCAGTGCAGGGTCCGAGGTATTCGCACTGGATACGACGTATAA Forward: GAGCTTTTGGCCCGGG
mmu-miR-672-5p	RT Primer: GTCGTATCCAGTGCAGGGTCCGAGGTATTCGCACTGGATACGACTCACAC Forward: CGCGTGAGGTTGGTGTACTGT
mmu-miR-7001-5p	RT Primer: GTCGTATCCAGTGCAGGGTCCGAGGTATTCGCACTGGATACGACATGCTC Forward: GAGGCAGGGTGTGAGCGT
mmu-miR-434-3p	RT Primer: GTCGTATCCAGTGCAGGGTCCGAGGTATTCGCACTGGATACGACAGGAGT Forward: CGCGTTTGAACCATCACTCG

### Target gene prediction of differentially expressed miRs

Differentially expressed microRNAs with more than a 2-fold change were used to pull predicted target genes from TargetScanMouse 7.1 (TargetScan.org) [42] and the miRanda database (microRNA.org) [43]. The common predicted target genes pulled from both databases were used to construct a regulatory network. The relationship strength, which indicates the network characteristic value, was calculated based on the functional microRNA position in the network. The microRNAs with the highest eigenvalues were placed at pivotal positions in the network, and these microRNAs bear the strongest regulatory ability.

### Gene ontology and pathway enrichment analyses

The predicted target genes were subjected to Gene Ontology (GO: http://www.geneontology.org) analysis to map the molecular and biological functional GO terms. A high enrichment score indicates that a GO term is more vulnerable to experimental factors. Kyoto Encyclopedia of Genes and Genomes (KEGG; http://www.genome.jp/kegg/) was explored to analyze the enrichments of functional pathways and networks. The interaction networks between significant pathways and differentially expressed miRNAs were constructed based on a two-sided Fisher’s exact test [44] and a χ^2^ test [45].

### Statistical analysis

SPSS 22.0 software (SPSS Inc., Chicago, IL, USA) was used for statistical analysis. All data, including Oil Red O stain, RT-qPCR, and lipid profiling were analyzed using a student’s *t* test [46], and presented as the mean plus or minus the standard deviation.

## Results

The whole spectrum of miRNA regulation during the process of atherosclerosis is still unclear. This study intends to identify more novel dysregulated microRNAs in atherosclerotic mice. The aim is to provide more research and therapeutic targets for atherosclerosis. We report here additional novel dysregulated miRNAs in ApoE-/- mice fed with a high-fat diet for 12 weeks and bioinformatic analysis on predicted target genes.

### High-fat diet apoE^-^/^-^ mice developed more significant atherosclerotic lesions

**Figure 1.**
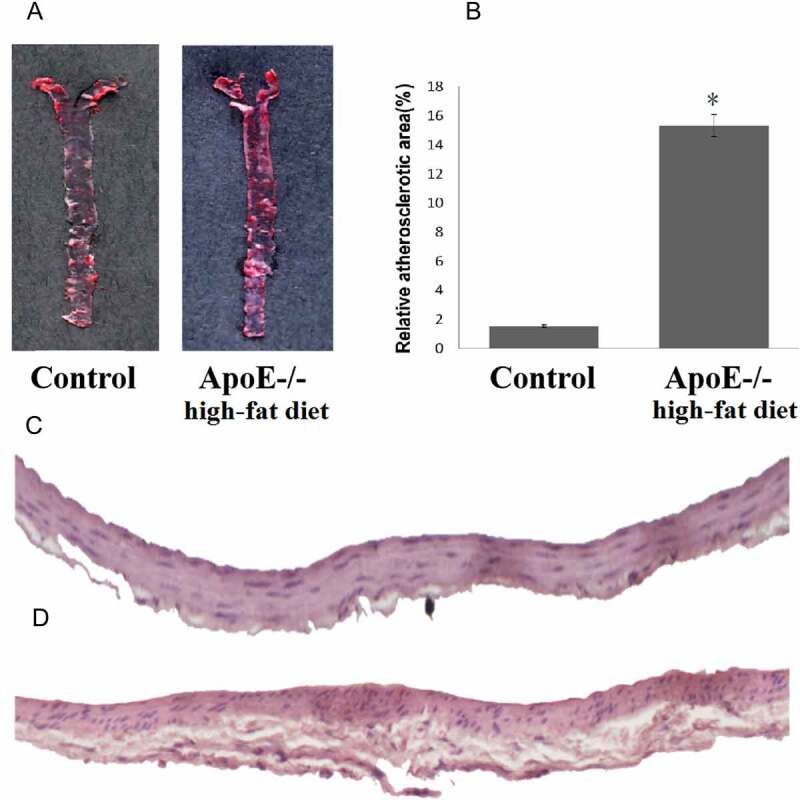
ApoE^-^/^-^ mice fed with a high-fat diet exhibit a more pronounced atherosclerotic plaque. A: Oil Red O staining of atherosclerotic lesions in ApoE-/- mice fed with a high-fat diet and control mice. B: Quantitative lesion areas (Oil Red O positive) are calculated as a percentage of total aorta surface and presented as the mean ± standard deviation of the mean (%), *p < 0.05; There were three mice in each group. C: H&E stained transverse sections of the aortic arch in control (100×). D: H&E stained transverse sections of the aortic arch in ApoE-/- mice fed with a high-fat diet (100×)

Compared to the control mice, ApoE-/- mice fed with a high-fat diet exhibited significant development of atherosclerotic plaques as demonstrated using Oil Red O stain. A wide distribution of atherosclerotic lesions was observed throughout the entire aorta tree in ApoE-/- mice fed with a high-fat diet, and limited lesions were observed in the aortic root area in control mice (Figure 1(A)). The area of aorta atherosclerotic lesions in ApoE^-^/^-^ mice fed with a high-fat diet was significantly higher than that of the control mice (15.32% vs 1.52%, p < 0.01, Figure 1(B)). H&E staining showed extensive lesions in ApoE^-^/^-^ mice fed with a high-fat diet (Figure 1(C-D)).

### ApoE^-^/^-^ mice fed with a high-fat diet exhibited hyperlipidemia with regular HDL-C

Serum lipid profiling showed significant hyperlipidemia in ApoE^-^/^-^ mice fed with a high-fat diet, with serum levels of TC, TG, LDL-C, and ox-LDL significantly increased compared to the control group, and there was no change in HDL-C (Table 2).

**Table 2.** ApoE^-^/^-^ mice fed with a high-fat diet exhibited higher levels of serum lipids except for HDL-C

	TC (mmol/L)	TG (mmol/L)	HDL-C (mmol/L)	LDL-C (mmol/L)	Ox-LDL (mmol/L)
Control	2.66 ± 0.31	0.79 ± 0.18	2.81 ± 0.24	7.47 ± 1.99	16.8 ± 1.90
ApoE^-^/^-^ high-fat diet	30.13 ± 4.25*	4.52 ± 0.76*	2.67 ± 0.19	14.67 ± 1.62*	30.67 ± 1.10*

*p < 0.05.

### Differentially expressed miRs and predicted target genes

**Figure 2.**
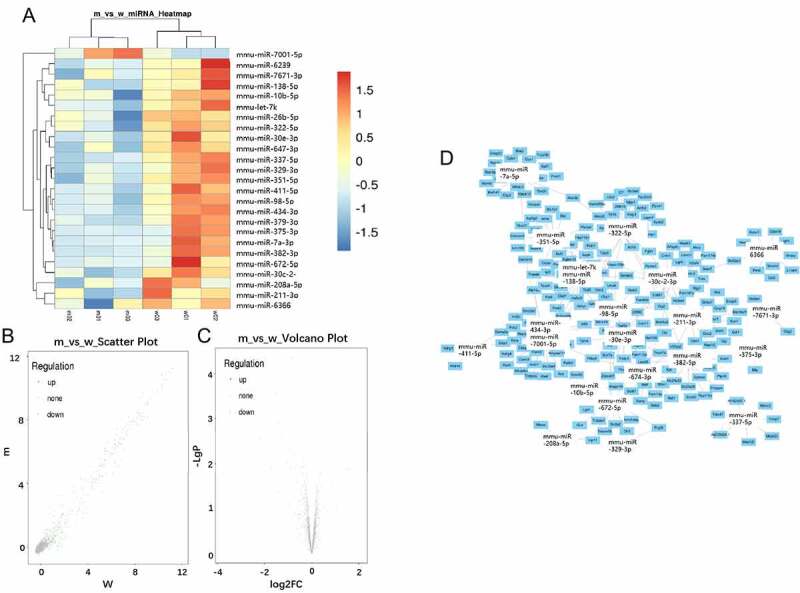
MiRNAs expression profiling using GeneChip analysis. A: Hierarchical cluster analysis of differentially expressed miRNAs (p < 0.05; n = 3). B: Scatter plot map of differentially expressed miRNAs. Upregulated miRNAs were marked in red, and downregulated miRNAs were marked in green (p < 0.05; n = 3). C: The volcano map overall gene expression. D: Predicted target genes of 24 differentially expressed miRNAs

Microarray data analysis with significant filters (fold change ≥2, p < 0.05) identified 25 differentially expressed miRs in ApoE-/- mice fed with a high-fat diet (Table 3), among which 24 miRs were downregulated, only mmu-miR-7001-5p was found to be upregulated. The most downregulated miR was mmu-miR-375-3p (fold change = -13.9). Hierarchical clustering analysis showed distinct expression patterns between two groups (Figure 2(A)). A scatter plot map and the Volcano plot map were also used to assess the differential expression patterns of microarray data (Figure 2(B-C)). A few cores differentially expressed microRNAs, and their respective target genes were shown (Figure 2(D)).

**Table 3.** Differentially expressed microRNAs with a high significance (p < 0.05,fold change≥2.0)

Probe set	Transcript.ID.Array.Design.	fold change	*P.Value*	Regulation
MIMAT0000739_st MIMAT0000747_st MIMAT0001422_st MIMAT0000743_st MIMAT0000677_st MIMAT0003741_st MIMAT0000150_st MIMAT0000534_st MIMAT0003735_st MIMAT0000609_st MIMAT0004644_st MIMAT0025110_st MIMAT0000545_st MIMAT0017014_st MIMAT0004747_st MIMAT0005438_st MIMAT0000548_st MIMAT0029849_st MIMAT0000249_st MIMAT0017059_st MIMAT0024860_st MIMAT0025580_st MIMAT0000208_st MIMAT0027904_st	mmu-miR-434-3p mmu-miR-382-5p mmu-miR-329-3p mmu-miR-379-5p mmu-miR-7a-5p mmu-miR-674-3p mmu-miR-138-5p mmu-miR-26b-5p mmu-miR-672-5p mmu-miR-351-5p mmu-miR-337-5p mmu-miR-6366 mmu-miR-98-5p mmu-miR-208a-5p mmu-miR-411-5p mmu-miR-30 c-2-3p mmu-miR-322-5p mmu-miR-7671-3p mmu-miR-30e-3p mmu-miR-211-3p mmu-miR-6239 mmu-let-7k mmu-miR-10b-5p mmu-miR-7001-5p	-13.93994 -5.961757 -3.988368 -3.381547 -3.308576 -3.262847 -3.262847 -3.105263 -2.968656 -2.816603 -2.770042 -2.676591 -2.664066 -2.608746 -2.446921 -2.438473 -2.392655 -2.367063 -2.298771 -2.111502 -2.056051 -2.032289 -2.027658 2.9272093	0.018375 0.002912 0.002183 0.006986 0.012732 0.001639 0.001639 0.01418 0.036304 0.003982 0.000285 0.040688 0.001186 0.015035 0.006458 0.00091 0.002247 0.02349 0.001399 0.006546 0.023371 0.00467 0.009782 0.0131507	down down down down down down down down down down down down down down down down down down down down down down down up

### GO enrichment analysis

**Figure 3.**
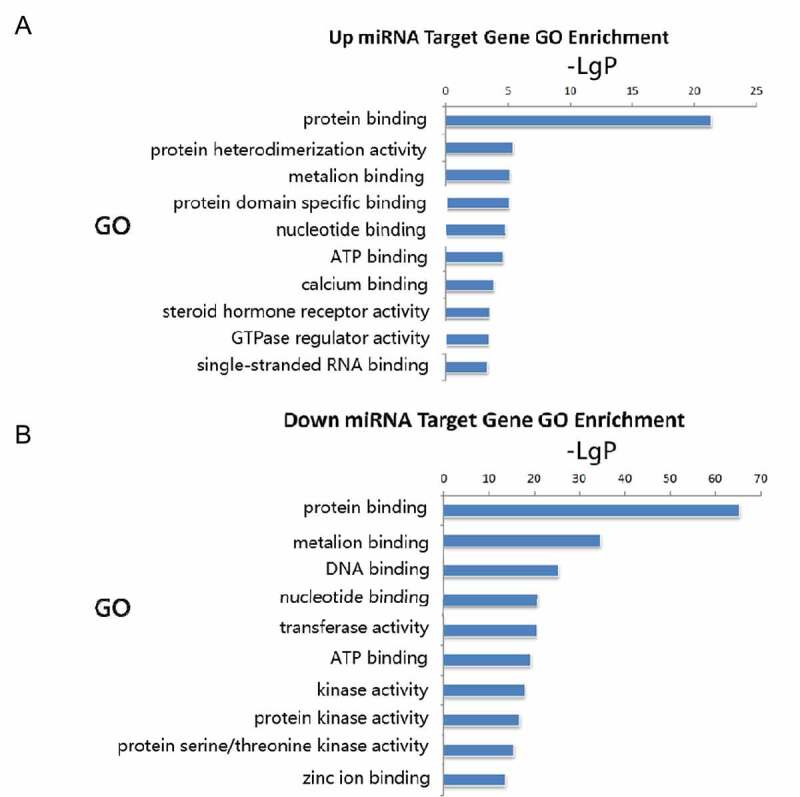
Gene ontology molecular function enrichment analyses of predicted target genes. A: Target genes of upregulated miRNA. B: Target genes of downregulated miRNAs

Common predicted target genes extracted from TargetScan and miRanda databases were subjected to GO analysis. The up-regulated miR target genes were found to be highly involved in protein binding, protein heterodimerization activity, metal ion binding, protein domain-specific binding, nucleotide-binding, and ATP binding (Figure 3(B)). Downregulated miRNA target genes showed significant enriched molecular functions in protein binding, metal ion binding, DNA binding, nucleotide binding, transferase activity, and ATP binding (Figure 3(B)).

### Pathway enrichment analysis

**Figure 4.**
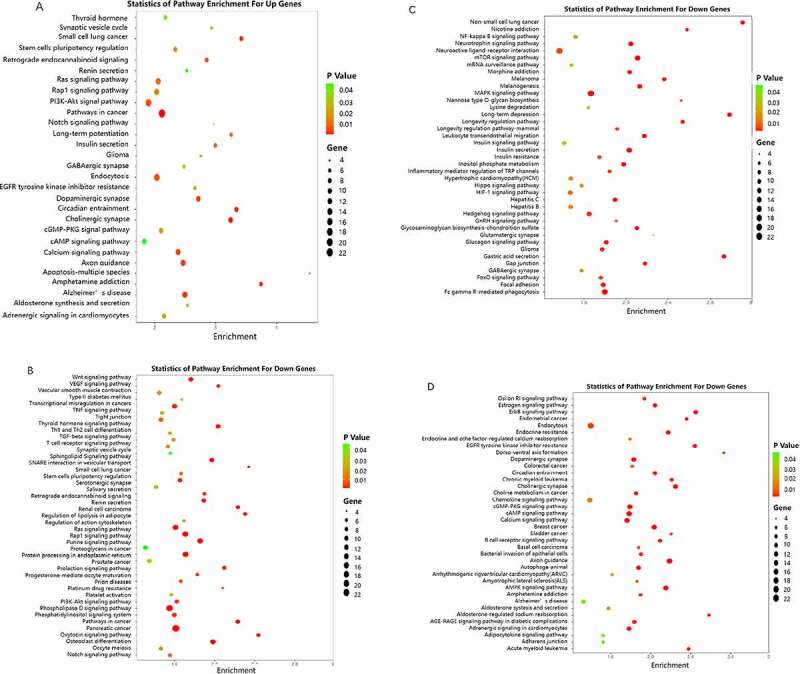
Pathway enrichment analyses of predicted target genes. A: Target genes of up regulated miRNAs. B-D: Target genes of down regulated miRNA

**Figure 5.**
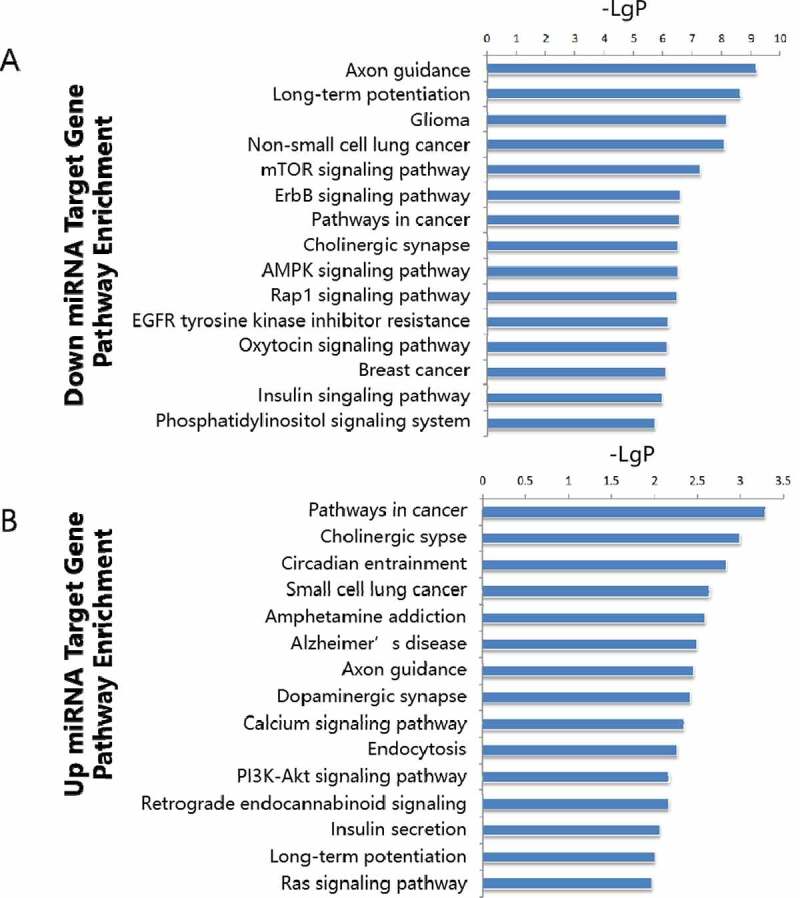
Summary of significantly enriched pathways. A: Target genes of downregulated miRNAs. B: Target genes of upregulated miRNA

A microRNA target gene pathway enrichment analysis helps to understand the metabolic pathways (Figure 4(A-D)). According to the statistical results, we found that the up-regulated miR target genes showed pathway enrichment in cancer, cholinergic synapse, circadian entrainment, small cell lung cancer, and amphetamine addiction (Figure 5(A)). Downregulated miR target genes showed pathway enrichments in axon guidance, long-term potentiation, glioma, non-small lung cancer, and mTOR signaling pathway (Figure 5(B)).

### RT-qPCR validation

**Figure 6.**
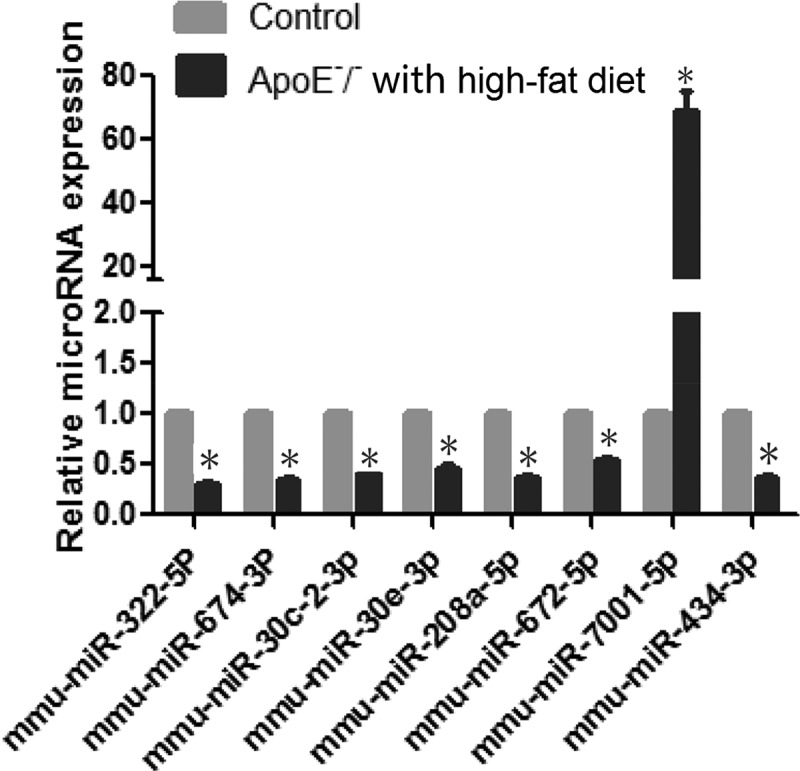
Real-Time qPCR validation of selected miRNAs. *p < 0.01

Eight differentially expressed miRs were selected for further RT-qPCR validation. RT-qPCR confirmed significant downregulation of miR-322-5p, miR-674-3p, miR-30 c-2-3p, miR-208a-5p, miR-672-5p, miR-434-3p, and miR-30e-3p, and upregulation of miR-7001-5p (Figure 6).

## Discussion

An ApoE-/- knockout mice model has long been used as an atherosclerogenic model which develops atherosclerosis lesions in the middle-to-large arterial tree. A Western-type diet or a high-fat diet significantly speeds up the atherosclerotic lesion and distribution [47,48]. In our study, after a 12-week high fat diet, ApoE^-^/^-^ mice fed with a high-fat diet exhibited a hyperlipidemia profile with increased serum TC, TG, LDL-C, and ox-LDL, and extensive atherosclerotic lesions on the aorta tree compared to the control group, which showed limited lesions in the aortic root and arch region.

Atherosclerogenesis is a prolonged chronic inflammatory process; the complexity of atherosclerosis involves endothelia dysfunction, inflammatory response, macrophage infiltration/polarization, vascular smooth muscle cells dedifferentiation, and lipid dysregulation. ApoE-/- mice fed with a high-fat diet are widely used for miRs profiling, regulatory, and therapeutic targeting studies related to atherosclerosis [35,49,36,39,50]. An increasing list of dysregulated miRs in the ApoE-/- mice with a high-fat diet model under different conditions reveals a dynamic, time, and stimuli dependent regulation spectra. With significant atherosclerotic lesions at 12 weeks, we identified a total of 25 significantly differentially expressed microRANs in ApoE-/- mice with a high-fat diet using GeneChip profiling, with most being downregulated (24 of 25) and one being upregulated. The most downregulated miRs identification is very similar to a report on rats fed with a high-fat and high-sucrose diet for 4 weeks [51], which found 28 out of 29 downregulated miRs. Among them, there are common miRs identified previously and newly identified miRs. Zhen identified a total of 28 dysregulated miRs at different stages of atherosclerosis in ApoE^-^/^-^ mice fed with a Western-type diet [36]. miR-434-3p was downregulated in both experiments. miR-434-3p plays a regulatory role in germ cell development [52] and skeletal muscle aging through DNA hypomethylation [53, Shang et al.]. Three downregulated miRs (miR-375-3p, miR-30e-3p, and miR-26b-5p) were also identified by another study on ApoE^-^/^-^ mice fed with a Western-type diet for 8 months [35]. The whole miR-375-3p was found upregulated in a previous study; it was downregulated in our study. Even though there is no specific report of miR-375-3p on atherosclerosis, it was reported to be upregulated in angiotensin II–induced primary cardiomyocyte hypertrophy rats [54]. Fu and his colleagues demonstrated that exosomal mmu-miR-375-3p was dramatically increased in the serum of an STZ treated mouse prior to the disturbance of blood glucose and insulin [55]. Knudsen considered miR-375 as a potential regulator of the enteroendocrine lineage [56]. Another researcher revealed that miR-375-3p negatively modulated osteogenesis, and the relevant products of miR-375-3p might be developed into potential molecular drugs [57].

MiR-382-5p was reported to be involved in the regulation of cholesterol homeostasis and inflammatory reactions [58] and in many other diseases like glioma cell proliferation, migration and invasion [59], breast cancer [60], glioma angiogenesis [61], acute promyelocytic leukemia (Liu et al. 2019a), and primary myelofibrosis [62]. Among another 5 downregulated miRs (miR-329-3p, miR-379-5p, miR-7a-5p, miR-674-3p, and miR-138-5p) with fold change less than 3 in this study, miR-329-3p was identified as upregulated in the progression of diabetic atherosclerotic rat [63], and miR-379-5p has been reported to play an important regulatory role in the proliferation and migration of many tumor cells [64–67]. There are no reports on the pathogenesis of atherosclerosis involving miR-7a-5p, miR-674-3p, miR-6239, miR-7671-3p, miR-674-3p, miR-138-5p, and miR-7001-5p. MiR-7001-5p is the only upregulated microRNA identified in this study, and its role in atherosclerogenesis is not clear.

Short non-coding miRNAs are gene silencers that exert biological effects through gene expression regulation of target genes participating in multiple signaling pathways notoriously involved in atherosclerogenesis, like phosphatidylinositol 3-kinase/ serine/threonine kinase 1/ mammalian target of rapamycin (PI3K/Akt/mTOR), mitogen-activated protein kinase (MAPK), Ras homolog gene family, member A/ (RhoA/ROCK), transforming growth factor-beta (TGF-beta), epidermal growth factor receptor (EGFR), Nuclear Factor-KappaB (NF-kappaB), Janus kinase 1/signal transducer and activator of transcription 1 (JAK1/STAT1), Notch and Wnt signaling pathways, etc [26,38,68–72]. In the present study, both the GO and KEGG pathway enrichment analysis identified high enrichment of target genes involved in. the PI3K/Akt/mTOR, Adenosine Monophosphate Activated Protein Kinase (AMPK)/MAPK, Wnt, Notch, insulin, and the NF-kappaB signaling pathways, and the PI3K/Akt/mTOR signaling pathway was identified in both upregulated and downregulated miRs target genes. Our data confirm the correct identification of more dysregulated miRs in atherosclerosis.

This study bears the limitation of one-time point sampling. Therefore, additional systematic experiments are needed to verify the mechanism of miRNAs on atherosclerosis.

## Conclusions

This study has identified 25 differentially expressed miRs, including a set of novel miRs involved in atherosclerogenesis using GeneChip microarray profiling of miRs in high-fat-fed ApoE-/- mice fet with a high-fat diet, and bioinformatic analysis reveals that the PI3K/Akt/mTOR signaling pathway is highly enriched in the predicted target genes [73]. The novel identified dysregulated miRs suggest a broad spectrum of miRs dysregulation in the progression of atherosclerosis and provide additional research and therapeutic targets [74].

## Highlights

- A total of 25 significant differentially expressed microRNAs were identified.
- In atherosclerosis, miR-701-5p expression is up-regulated.
- The PI3K/Akt/mTOR signaling pathway is highly involved in atherosclerosis.
